# Operational considerations for implementing culture-free mycobacterial sequencing in routine laboratory settings

**DOI:** 10.1128/jcm.00509-26

**Published:** 2026-06-12

**Authors:** Gabriel Rojas-Ponce

**Affiliations:** 1 Global TB-Lab Operations Group (TB-LOG), Edmonton, Alberta, Canada; University of Western Australia, Perth, Australia

**Keywords:** whole genome sequencing, molecular diagnostic, tuberculosis, TB culture

## LETTER

I read with interest the study by Hashimoto et al. (1), which presents a culture-free sequencing approach for subspecies-level identification of mycobacteria directly from sputum. The authors demonstrate promising performance, particularly in smear-positive specimens, and highlight the potential of such workflows to accelerate diagnostics.

In line with the authors’ acknowledgment that these findings may not be generalizable to diverse clinical settings or patient populations, I would like to complement their work by highlighting operational aspects relevant to paucibacillary samples and future studies.

The proposed workflow includes a mucolysis step in which each sputum specimen is mixed with approximately three times its volume of a semi-alkaline protease solution. While effective under experimental conditions, this ratio may present scalability challenges in high-throughput settings, raising questions about routine implementation.

The mucolysis step is described as being performed with “gentle agitation,” yet the mode of mixing is not defined. In practice, different approaches (e.g., tube rotation, orbital shaking, or manual inversion) may yield varying degrees of homogenization. Given the heterogeneous nature of sputum, such variability may influence reagent exposure and downstream performance.

Following centrifugation, the pellet is resuspended in phosphate-buffered saline (PBS), although ambiguity remains regarding whether this refers to a defined final volume or the addition of a fixed volume. This distinction directly affects the concentration of mycobacteria and DNA available for downstream analysis. Buffer composition and pH are also not consistently specified.

The decontamination step using N-acetyl-L-cysteine–sodium hydroxide (NALC–NaOH) is a critical component of the workflow, yet key parameters, such as NaOH concentration, exposure time, and sample-to-reagent ratios, are not explicitly defined. In routine practice, these variables vary across laboratories and may significantly influence decontamination efficiency, mycobacterial viability (2), and DNA integrity (3). Given that mucolysis is already performed using a protease-based pretreatment, it remains unclear whether NALC is necessary or introduces redundancy, with implications for workflow simplification.

The use of a shared pretreatment workflow for both culture and culture-free sequencing is methodologically sound, as it avoids biases introduced by splitting heterogeneous samples. However, both downstream analyses depend on the same recovered pellet. Under these conditions, washing steps, pellet recovery, and aliquoting become critical determinants of representativeness. Operator-dependent steps, such as supernatant removal, centrifugation format, and tube volume, may influence material recovery. These factors are likely to have limited impact in smear-positive specimens but become increasingly important in low-burden samples, where small variations may affect both culture recovery and sequencing sensitivity. Representativeness may also be relevant in the context of follow-up of patients under treatment or mixed mycobacterial populations. Such heterogeneity has been described in the context of genetic variability and resistance interpretation in *Mycobacterium tuberculosis* (4).

The physical characteristics of sputum may further influence pellet formation, recovery, and consistency following decontamination and centrifugation, affecting downstream aliquoting and analytical sensitivity.

Taken together, these observations highlight that culture-free sequencing approaches remain closely linked to pre-analytical and operational factors. I suggest that future studies explicitly report key processing parameters and evaluate workflows under routine laboratory conditions to ensure reproducibility across clinical settings.
